# SARS-CoV-2 transmission in classroom settings: Effects of mitigation, age, and Delta variant

**DOI:** 10.1063/5.0067798

**Published:** 2021-11-09

**Authors:** Aaron Foster, Michael Kinzel

**Affiliations:** University of Central Florida, Mechanical and Aerospace Engineering, Orlando, Florida 32766, USA

## Abstract

Traditional, in-person classroom settings have been limited during the COVID-19 pandemic due to their potential to transmit severe acute respiratory syndrome coronavirus 2 (SARS-CoV-2) among students, teachers, and other educational workers. Using computational fluid dynamics simulations, mitigation strategies that span approaches using face coverings, various ventilation schemes, air purifiers/cleaners, and desk shields are systematically evaluated in thermally controlled classrooms. Individually, face coverings and source control were the most effective, which was followed by well-designed ventilation systems. The use of desk shields was also studied and appeared to be ineffective. The best mitigation approach is shown to be through multiple measures—using face coverings and ventilation systems combined with air purifiers. The studies were extended to elementary schools and consider Delta variants of SARS-CoV-2. In elementary settings, the reduced pulmonary and viral emission rates of small children are observed to drive reduced transmission rates, to values even lower than those observed with several mitigation methods for classrooms with adults. The Delta variant, with adults, was evaluated by considering an increase in quanta and indicated higher transmission probabilities. These increases are levels that are controllable by increasing the mitigation methods. Results indicate several plans of action for schools to return to in-person schooling in the context of age and new variants.

## I. INTRODUCTION

Educational systems are critical for societal function as they are foundational to train next-generation professionals, create a social setting critical for human development, as well as provide a means for parents and guardians to work. The COVID-19 pandemic has challenged these systems and their underlying functions to the society. School responses throughout the world during the pandemic included a variety of actions and interventions during the pandemic. These included no change whatsoever, to those using interventions and partial shutdowns, as well as complete shutdowns. The process of transitioning from partial and complete school shutdowns to normalcy with safe school operation demands systematic studies of various mitigation strategies. This goal demands developing a detailed understanding of the effectiveness of each mitigation strategy.

A driver of airborne transmission of severe acute respiratory syndrome coronavirus 2 (SARS-CoV-2) is associated with inhaling an infectious dose of viral particles. Such a process involves the probabilistic events of inhaling small dispersed viral particles.^1^ The primary drivers include respiratory rates and the local concentration of virus in the air.^2^ Controlling the respiratory rate would imply activity level reduction, but it is also a factor that relates to age. The local concentration, on the other hand, is a much more controllable factor that relates to a combination of air circulation within a room, filtration/dilution, and reducing viral-particle emission. The airflow circulation within a classroom is highly variable as it is largely driven by the heating, ventilation and air conditioning (HVAC) systems. HVAC systems can range from no ventilation (e.g., baseboard heating) to those having a driven mixing capability (fans, ducting design, flow rates). These HVAC systems also have filters with variable efficiencies. High-end, high-efficiency particulate absorbing (HEPA) filters are associated with aircraft and air purifiers that filter over 99.9% of fine particulate. Lower grades are variable and common to commercial and residential settings and are typically rated using the minimum efficiency reporting value (MERV) standard. In general, these filters aim to reduce the buildup of dust, but these can also remove small viral particles. Although they are similar in that they both reduce buildup, filtering, and dilution with clean, ambient air, they are fundamentally different; filtering reduces viral particles by capturing them, while dilution reduces their concentration through mixing with air that is clean of the viral particles. Of similar interest are the individual factors, which include the usage of face coverings, social distancing, barriers, and other tools that alter the emission of viral particles. There are many studies indicating the importance of all these various factors; however, this effort adds importance in the context of systematic variation, usage in schools, and using precision methods to directly evaluate their importance.

Mitigation strategies have been broadly covered in previous studies. There are several studies indicating the importance of masks in terms of reducing droplets;^3–6^ time-of-stay in rooms;^7,8^ maximizing fresh air;^9–11^ student pods; noise reduction; symptom checking; and HVAC control among many others.^12^ These studies indicate a range of useful tools, but they lack detailed quantification of the mitigation methods. A recent study provided highly relevant insight into factors driving transmission within in-person classroom settings.^13^ Several key takeaways are that multiple measures become less effective, teacher masking is critical, and desk shields lead to an increased transmission probability. These studies directly highlight statistical trends that can be useful for future guidance; however, the strategy to safely reopen school may differ by school depending on local variations (temperature, building condition, etc.). This brings in a need to systematically study forms of airborne pathogen mitigation within schools.

Such real-world difficulties can be understood using computational fluid dynamics (CFD) to directly assess transmission in a controlled sense and within the context of complex scenarios. CFD is distinct from modern forms of Wells–Riley methods,^14^ as it can consider higher levels of HVAC design details, variability within various transmission paths in classroom environments, and improved accuracy by avoiding the well-mixed assumption.^15^ Through this platform, a systematic study of various classroom settings in combination with various mitigation strategies can yield additional insight into mitigation strategies.

CFD has been a useful tool to quantify transmission processes in confined spaces. Among other scenarios, it has been applied to restaurant settings,^16^ elevators,^17^ and classroom settings.^14,18^ The present effort performed CFD simulations using Star CCM+ (version 15.02.009) and includes the computation of transmission probability for 18 in-person classroom settings in a broad extension of our previous study^14^ and expands these data to analyze 2790 transmission routes to identify various mitigation strategies. The present results evaluate four variations in the HVAC design, three variations in the air purifiers,^19^ two variations in the classroom shape, effect of the Delta (B.1.617.2) strain, barriers, face covering, social distancing, emission rate of viral particles, and source control concepts.^20^ Each of these classrooms has nine students and one teacher and is evaluated for one hour. In the context of each scenario, we can assume that when any individual is infected, it leads to 90 possible transmission paths that can be analyzed statistically for each setting. The models also represent differences in the viral emission rates and breathing rates of adults compared to children. This allows us to compare different demographics in both elementary schools and university environments. The data are interpreted to indicate the effects of systematic variations in the room.

## II. METHODS

### A. Computational approach

The approach is based on a formulation within a finite-volume method, commercial CFD code, Star CCM+ (version 15.02.009). The method locally discretizes the classroom and is used to calculate mass, momentum, and energy conservation coupled to a quanta-dispersion equation (QDE).^14^ The QDE is an advection equation of viral-particle concentration, given by

$$\frac{\partial Q_{C}}{\partial t}+\nabla \cdot \left(Q_{C}\bar{V}\right)=0,$$ (1)

which is associated with the viral particles transported with aerosols emitted from various persons. Here, $Q_{C}$ is the quanta concentration and $\bar{V}$ is the fluid velocity vector. There are underlying assumptions of no slippage with the fluid velocity and no diffusion that, respectively, imply the method is specific to aerosols and is conservative in that it predicts a maximum concentration. The source for $Q_{C}$ occurs through boundary conditions at the mouth that result from breathing combined with emission rates of quanta. A summary of the values used for adults and children is provided in Table I (discussed in detail later). The QDE advection equation transports the quanta (or viral particles) throughout the room while accounting for various geometries (such as desk shields) and/or filtration in HVAC. The flow model is based on an ideal gas with an assumed constant pressure coupled to energy to drive thermal-related buoyancy from warm exhaled air and heating around the students/teachers. A detached eddy simulation (DES)^21^ approach was used to simulate aerosol dispersion due to turbulence. Some key underlying assumptions are as follows: (1) each person is emitting speaking rates of quanta and gas during exhalation (a conservative assumption); (2) participants are stationary (which leads to uncertainty); (3) the aerosols do not settle onto the floor (we focus on aerosols smaller than 10 *μ*m); (4) face coverings are all well fitted and consistent with surgical masks; and (4) each individual has a mask in a fixed position with a typical fit. We did not consider partial masking in the model. A more complete documentation of the methods is available in the work of Foster and Kinzel.^14^ This work is largely an expansion of the previous work to various configurations.

**TABLE I.** Breathing rate and viral emission rates for adults and children (ages 6 to 11).^24^

Description	Breathing rate $\left(\frac{m^{3}}{h}\right)\;$	Viral emission rate $\left(\frac{\text{quanta}}{h}\right)$
Adult	0.96	142
Children	0.29	29.6

### B. Transmission prediction

**FIG. 1.**
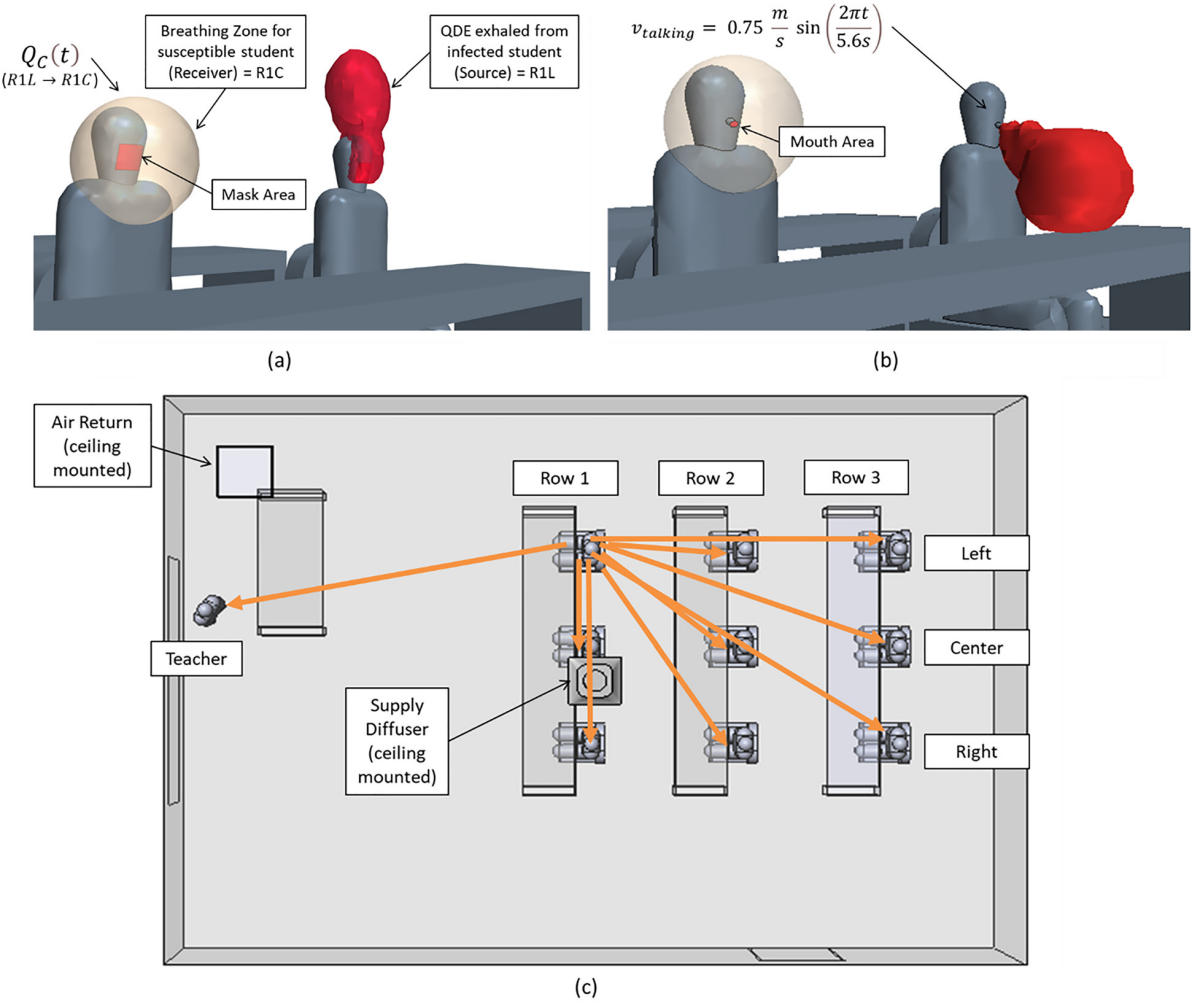
Breathing zones and source emission scenarios. (a) Scenario where face coverings are utilized. (b) No face covering is utilized, and (c) transmission paths (and distances) originating from the student in left side of row 1.

The present efforts convert the inhalation of viral particles based on the prediction of $Q_{c}(t)$ near each person in the classroom. Such a CFD-based infection probability demands special treatment. Within the CFD model, a breathing zone is defined in Fig. 1 where the QDE provides the local quanta concentration from the other occupants in the classroom. As indicated in Fig. 1(a), the condition with a face covering leads to a high quanta concentration region that is trapped in the thermal plume of the person exhaling the viral particles. Alternatively, the person without the face covering shown in Fig. 1(b) indicates a plume that traverses from the student and breaks through the thermal plume. The buildup of these quanta is tracked over time to provide a transmission probability. The infection-susceptibility model is investigated for all possible transmission routes to determine statistical risk, as well as dependencies on both the location and distance from infected individuals. The network of transmission routes^14^ consists of a scenario when any person is infected with respect to the others in the room as indicated in Fig. 1(c). Hence, each space with a total of *n* persons has the potential to transmit to n − 1 others (or n − 1 transmission routes). Each of these transmission routes has a different distance, which is indicated by the length of the arrow [Fig. 1(c)]. Considering the overall system, a total number of transmission routes of $n\times \left(n-1\right)$ are possible. In the context of the classroom, this leads to 10×9 or 90 transmission paths. Using such a detailed model enables the understanding probability of transmission events as well as distance and effect of various mitigation strategies. Additional details of the model settings and transmission routes are provided in the work of Foster and Kinzel.^14^

As previously observed,^14^ an efficient method to identify local quanta concentration is to define a breathing zone concept. In the context of the breathing zone as indicated in Fig. 1, here, the concentration inhaled from a susceptible person is assumed to occur from this region; hence, temporal integrals of the product of an assumed breathing volumetric flow rate and the breathing-zone concentration provide an estimate of the inhaled quanta. The result can be used to calculate infection probability from CFD analysis.

Transmission implies that one inhales an infectious dose of quanta exhaled from an infected person. We define this infectious dosage in terms of a quanta volume, $V_{\text{quanta}}(m^{3})$, which is defined as

$$V_{\text{quanta}}=\frac{p}{q}.$$ (2)

Here, *p* is the pulmonary rate for the susceptible and *q* is the quanta generation rate, which has values specified in Table I for adults^22^ and children.^23^ This quanta volume physically represents the volume of exhaled air that would need to be inhaled for a susceptible person to be infected. Based on previous studies indicating aerosol reduction in a human sneeze,^20,24^ it is possible to develop source control concepts that reduce the emission of quanta. In a sneeze for a single person, over a 95% reduction was observed. To be conservative, a quanta reduction value of 48.9% is assumed for a mitigation approach. From CFD analysis, the spatiotemporal variation of the exhaled $Q_{C}$ is directly computed within the breathing zone of the other individuals (Fig. 1). Additionally, as the viral particles are circulated through the room, there is a reduction within the air handling system with various grades of filters as well as through air purifiers. With these data, we can calculate the inhaled volume over time as

$$V_{\text{inhaled}}\left(t\right)=\int_{0}^{t}pQ_{C}\left(t\right){\left(1-\eta_{\text{mask}}\right)}^{2}dt,$$ (3)

where *p* is the pulmonary ventilation rate ( $m^{3}/h$), $Q_{C}\left(t\right)$ is the CFD scalar concentration over time, and *η_mask_* is the mask filtering efficiency (44%). Note that mask filtration is accounted for in both the exhale and inhale events. In this study, $Q_{C}\left(t\right)$ is the exhaled air concentration for an assumed single infected person in the classroom. This concentration could be used to represent any number of infected individuals in the space by summing together multiple passive scalars from multiple infected individuals. For this study, the current prevalence rates suggest that it is unlikely that there would be multiple infected students in a small classroom but would be a relevant comparison for larger venues.

From the quanta and inhaled volumes, we can calculate the fraction of an infectious dose and use a Poisson distribution to provide a Wells–Riley-equivalent calculation as follows:

$$P\left(t\right)=1-e^{\left(-\frac{V_{\text{inhaled}}\left(t\right)}{V_{\text{quanta}}}\right)},$$ (4)

where *P* is the probability of infection after a given exposure time (t). In these studies, a one-hour time frame is used for all calculations.

### C. Classroom configurations and mitigation methods

**FIG. 2.**
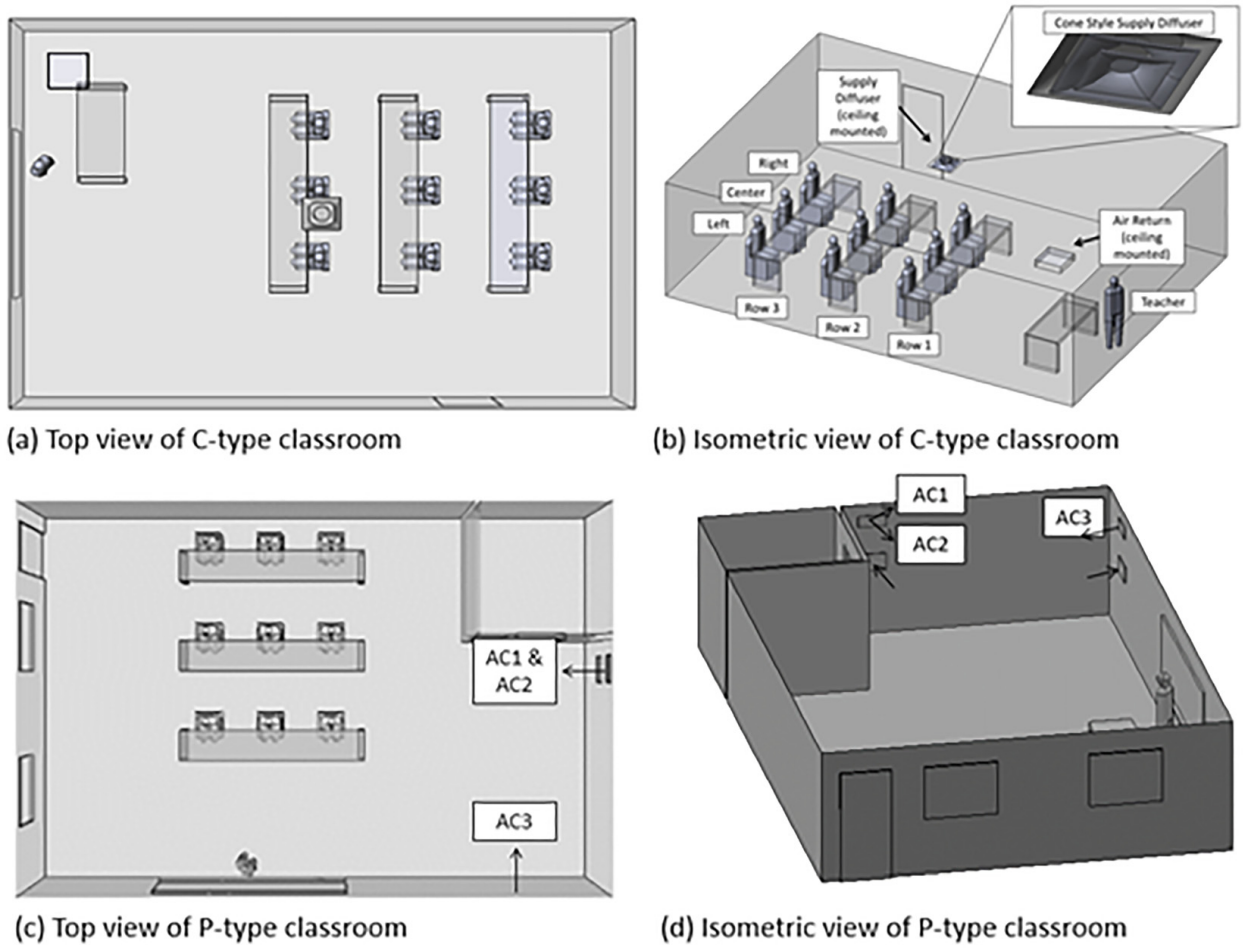
Classroom settings with nine students and one teacher. Students are not social distancing. These models account for HVAC details with common filters as well as interactions with the persons, desks, and other features. Figures (a) and (b) indicate the baseline, C-type classroom while figures (c) and (d) indicate a portable, P-type classroom.

**FIG. 3.**
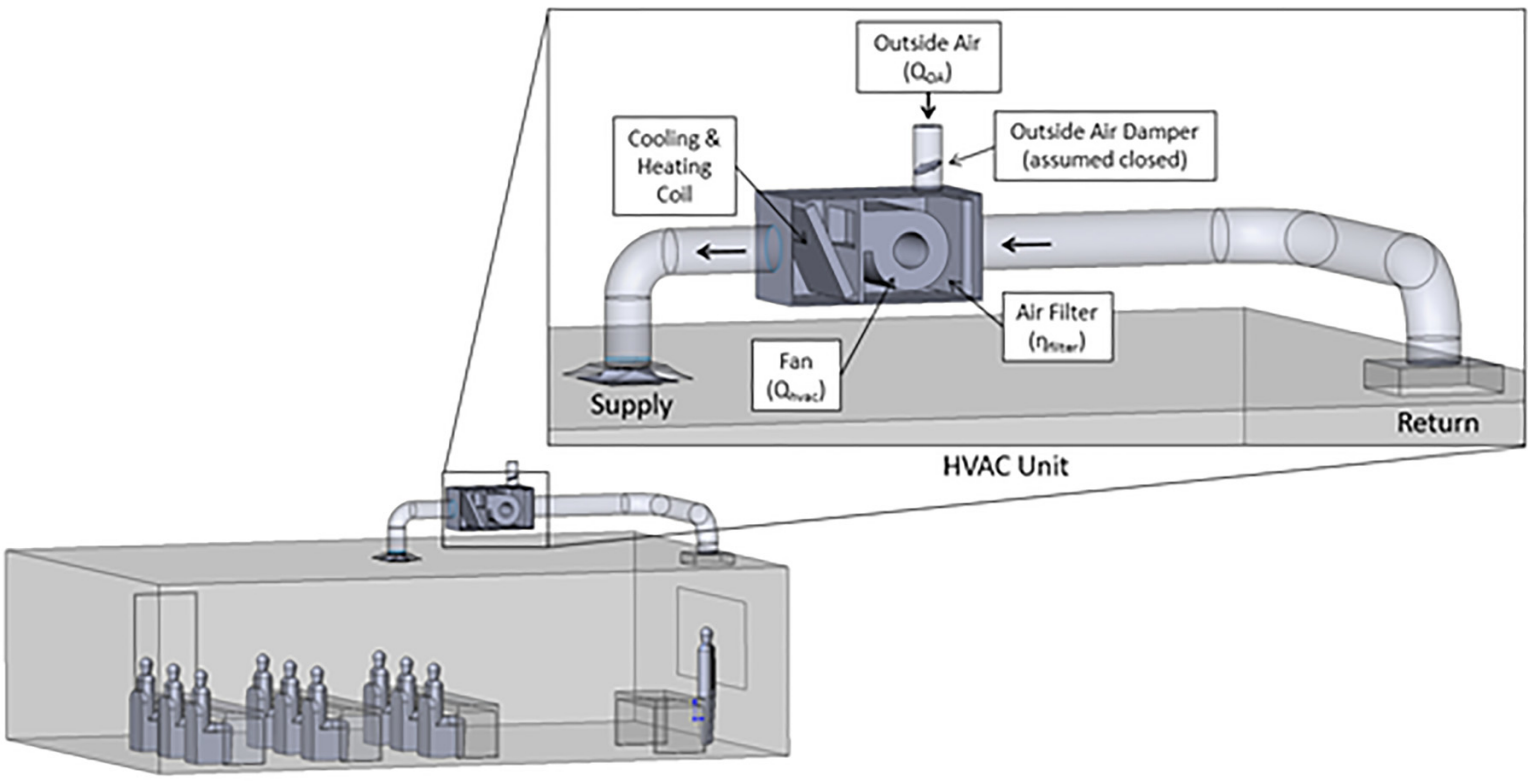
Diagram of the HVAC systems evaluated in the present work.

The above equations are solved in the context of two typical classroom configurations. The present effort studies a C-type classroom which is intended to represent a conventional classroom configuration and size and is depicted in Figs. 2(a) and 2(b). A goal was to evaluate if a portable classroom would be more prone to transmission events than a conventional one due to poor mixing and circulation from the HVAC system. Hence, we developed a model of a portable classroom configuration, P-type, which is depicted in Figs. 2(c) and 2(d). The overall arrangement of the HVAC system is depicted in Fig. 3. Note that there are filters within the ductwork or HVAC unit, purifiers within the classroom, and filters bringing in fresh ambient air from outside (subscripted with supp). This work focuses on a conservative scenario where fresh air is not introduced to the classroom ( $Q_{\text{supp}}\to 0$), which is consistent with times associated with cold and warm weather, which yield thermal losses/gains. Additionally, the bulk turbulence intensity in the room that drives mixing is accounted for through the multiscale DES turbulence model; note that a turbulent viscosity ratio of ten is assumed for all inlets (mouths, inlet vents, and air purifiers). These scenarios are proposed to develop a broader insight into classroom transmission events.

The boundary conditions applied to these classrooms are consistent with common values associated with heating, body temperatures, breathing and ventilation rates, etc. The inputs are summarized in Tables II and III. The filtration rates for the MERV 7 and 11 filters are provided from Azimi and Stephens.^25^ Note that the P-type classroom has a higher ventilation rate than the C-type classroom; however, the MERV rating is lower. The body temperatures are consistent and both cooling and heating configurations are considered.

**TABLE II.** Summary of input conditions for typical classroom. Inputs span approaches relevant to face coverings and source control, supply type, wall boundary conditions, and body temperature.

Description of conditions	Type	Flow	Pressure	Temperature	Quanta concentration
Face covering	Velocity inlet	$3.2\times 10^{-4}\;\frac{m^{3}}{s}$(0.67 cfm)	N/A	35 °C (95 °F)	142 Quanta/h (adults) 29.6 Quanta/h (children)
No face covering	Velocity inlet	$0.75\;\frac{m}{s}\;\text{sin}\left(\frac{2\pi t}{5.6\;s}\right)$	N/A	35 °C (95 °F)	142 Quanta/h (adults) 29.6 Quanta/h (children)
No face covering and saliva-based source control	Velocity inlet	$0.75\;\frac{m}{s}\;\text{sin}\left(\frac{2\pi t}{5.6\;s}\right)$	N/A	35 °C (95 °F)	(1−0.489)142 Quanta/h (adults)
Supply vent	Velocity inlet	Classroom: 360 cfm Portable: 800 cfm	N/A	Heating: 32.2 °C (90 °F) Cooling: 15.6 °C (60 °F)	Classroom (MERV 11) $\left(1-0.72\right){\;\left.\bar{Q_{C}\left(t\right)}\right|}_{\text{RetVent}\;}$Portable (MERV 7) $\left(1-0.44\right){\;\left.\bar{Q_{C}\left(t\right)}\right|}_{\text{RetVent}\;}$
Return vent	Pressure outlet	N/A	1 atm	N/A	N/A
Walls and ceiling	Wall	N/A	N/A	No ventilation: 23.9 °C (75 °F) Cooling: 26.7 °C (80 °F) Heating: 21.1 °C (70 °F)	N/A
Bodies	Wall	N/A	N/A	29.4 °C (85 °F)	N/A
Heads	Wall	N/A	N/A	33.3 °C (92 °F)	N/A
Air initial condition	Fluid	N/A	N/A	23.9 °C (75 °F)	N/A

**TABLE III.** Summary of mitigating measures. Inputs span options for masking and source control, HVAC (flow rates/filtration), shielding, and air purifiers.

Classroom types	
C = Conventional classroom: 360 cfm; 3.4 ACH	P = Portable classroom: 800 cfm; 7.0 ACH
Masks and source control	
M = Surgical style disposable mask (44% equivalent filtration)	SSC = Saliva-based source control (48.9% reduction)
Ventilation	
H = Heating	C = Cooling
Filtration	
H = MERV 11 filter (72% equivalent filtration)	L = MERV 7 filter (44% equivalent filtration)
Desk shields	
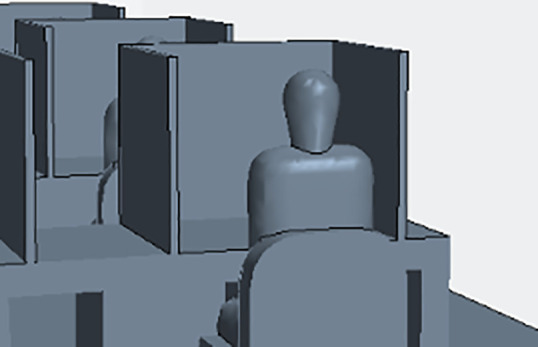	
Air purifiers	
A = 1 × 140 cfm; B = 2 × 140 cfm (located in center of room) 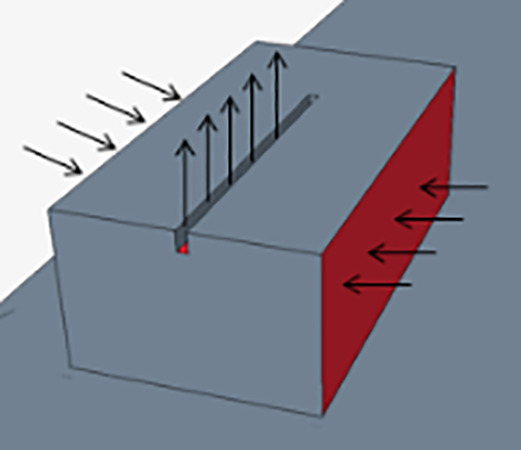	C = 1 × 280 cfm (located in rear corner of room) 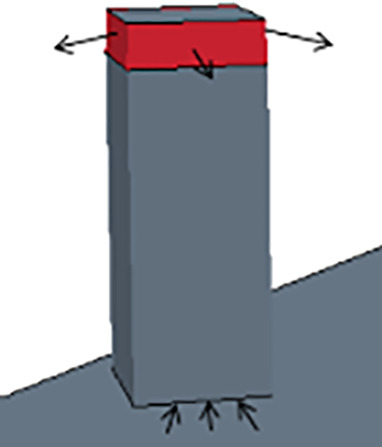

Mitigating measures include variations in the HVAC design (flow rates and filters), usage of air purifiers, and source control measures, such as using face coverings or other source control measures, which are summarized in Table III. Source control configurations include surgical-style face coverings (M) and novel methods associated with foods that reduce aerosols, or saliva-based source control (SSC). Note that these foods have been found to naturally reduce aerosol generated during human sneezes.^20^ The ventilation scenarios include hearing (H) and cooling (C) for both types of classrooms. Portable classrooms typically have HVAC units with increased air changes per hour (ACH) values, while conventional classrooms typically are configured with lower ACH units. The filters in these classrooms range from high efficiency (H) MERV 11 filters standard to conventional classrooms, to lower efficiency (L) MERV 7 filters standard to portable classrooms. Additionally, desk shields are included in the model and evaluated. Finally, various strategies of applying air purifiers in these configurations are also evaluated in three configurations listed in Table III where A has one air purifier based on the clean air curtain model,^19^ B has two clean air curtain air purifiers doubling the rate, while C has a single, conventional, air purifier that has double the capacity of the clean air curtain. These mitigation methods are studied in the context of the various CFD models.

## III. RESULTS AND DISCUSSION

### A. College classroom

**FIG. 4.**
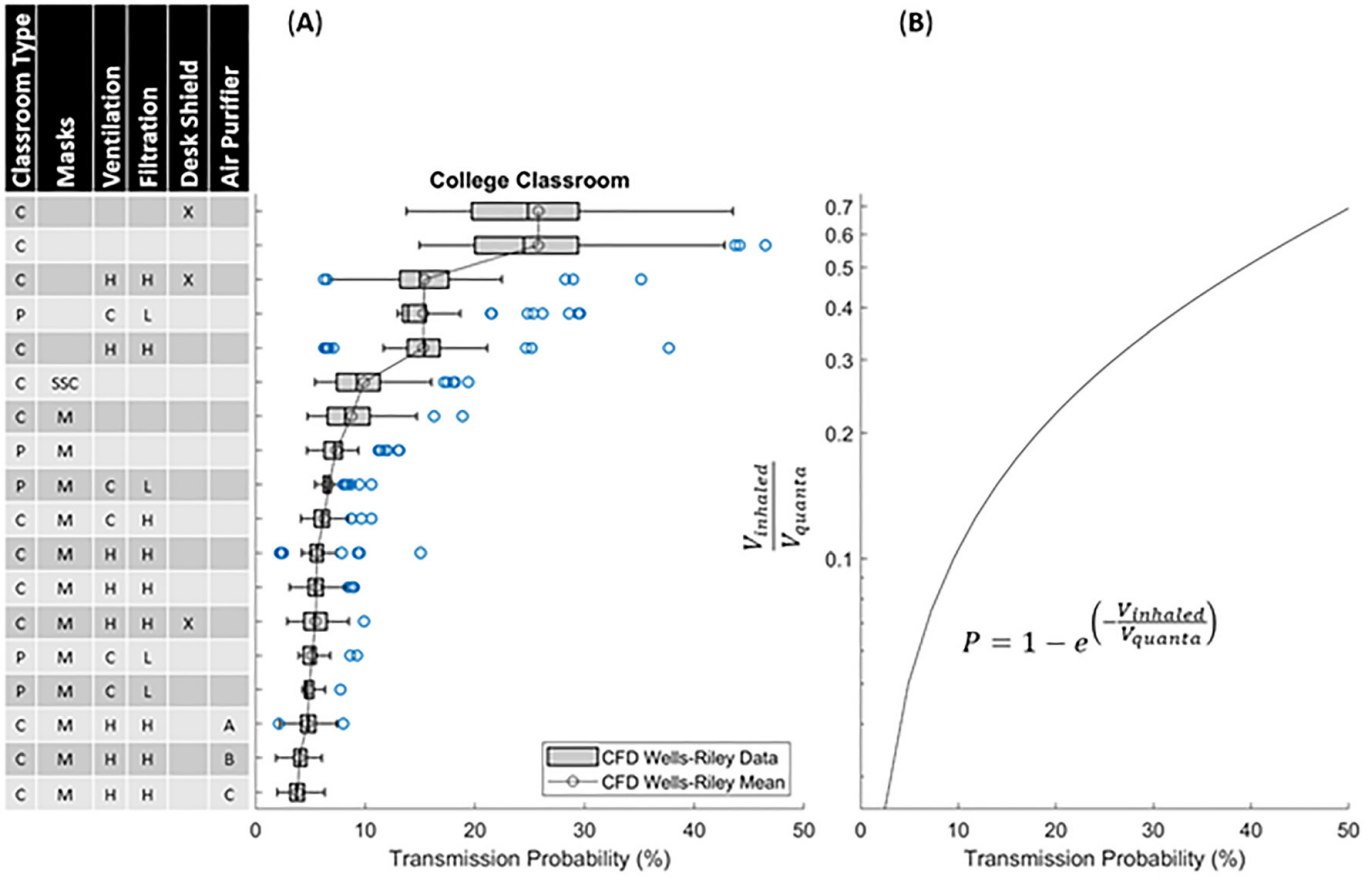
Predicted transmission probability in a one-hour college-level classroom setting with one infected person. (A) Statistical representation of the 90 different transmission routes for each condition. Note that the box–whisker plot uses the box to indicate the lower quartile, median, and upper quartile, while the bars indicate the lower and upper standard deviations. The blue dots indicate outliers, while the black dots with the line indicate the mean for each case. (B) Indication of the main driver being the ratio of volume inhaled to quanta concentration.

**FIG. 5.**
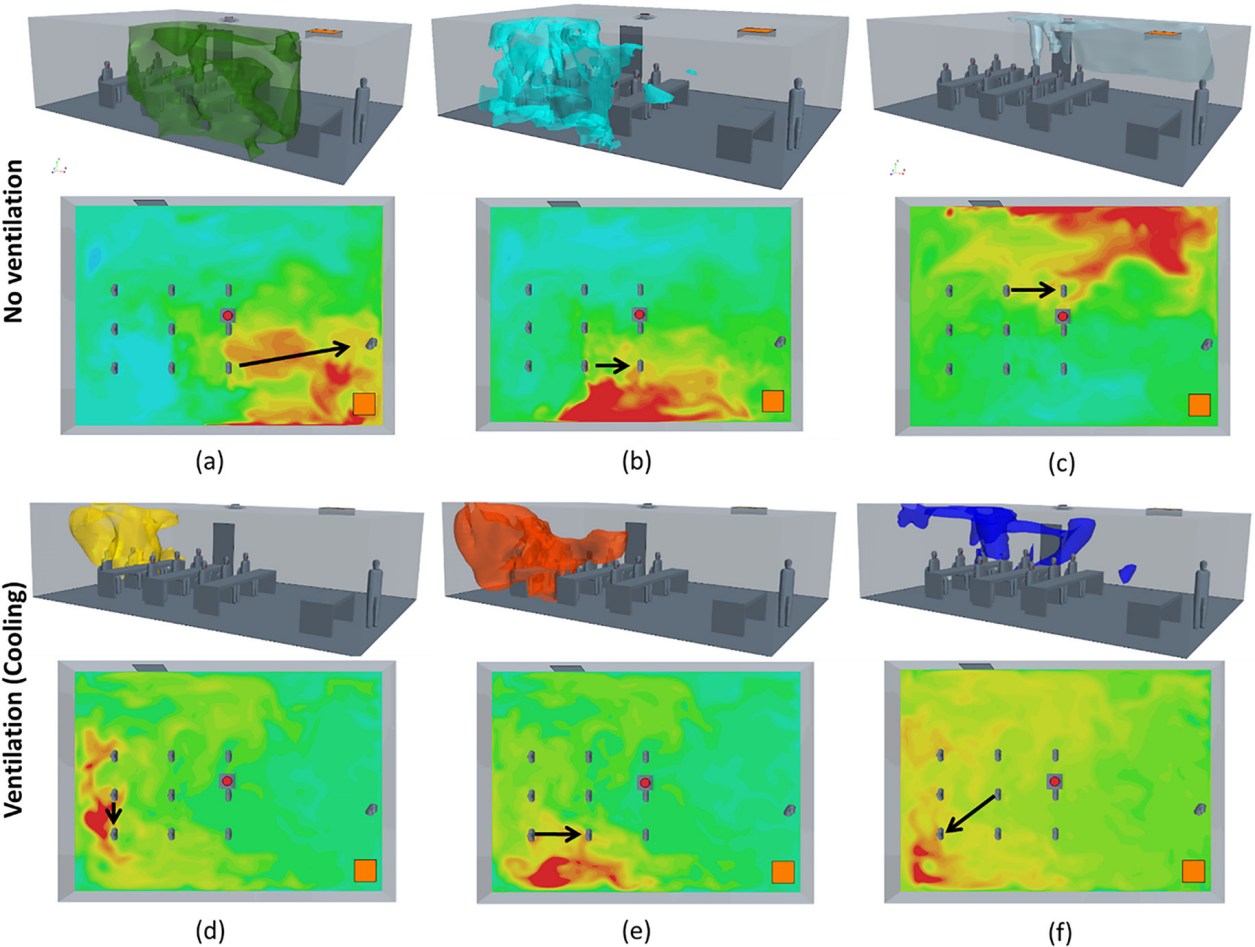
Quanta concentration values of the highest, second highest, and third highest transmission probability routes for the no ventilation [(a)–(c)] and ventilated with cooling [(d)–(f)] cases after 60 min. Each case plots the 3D QDE values (upper) along with contour maps of quanta concentration at the elevation of students' heads along with the route of interest highlighted.

**FIG. 6.**
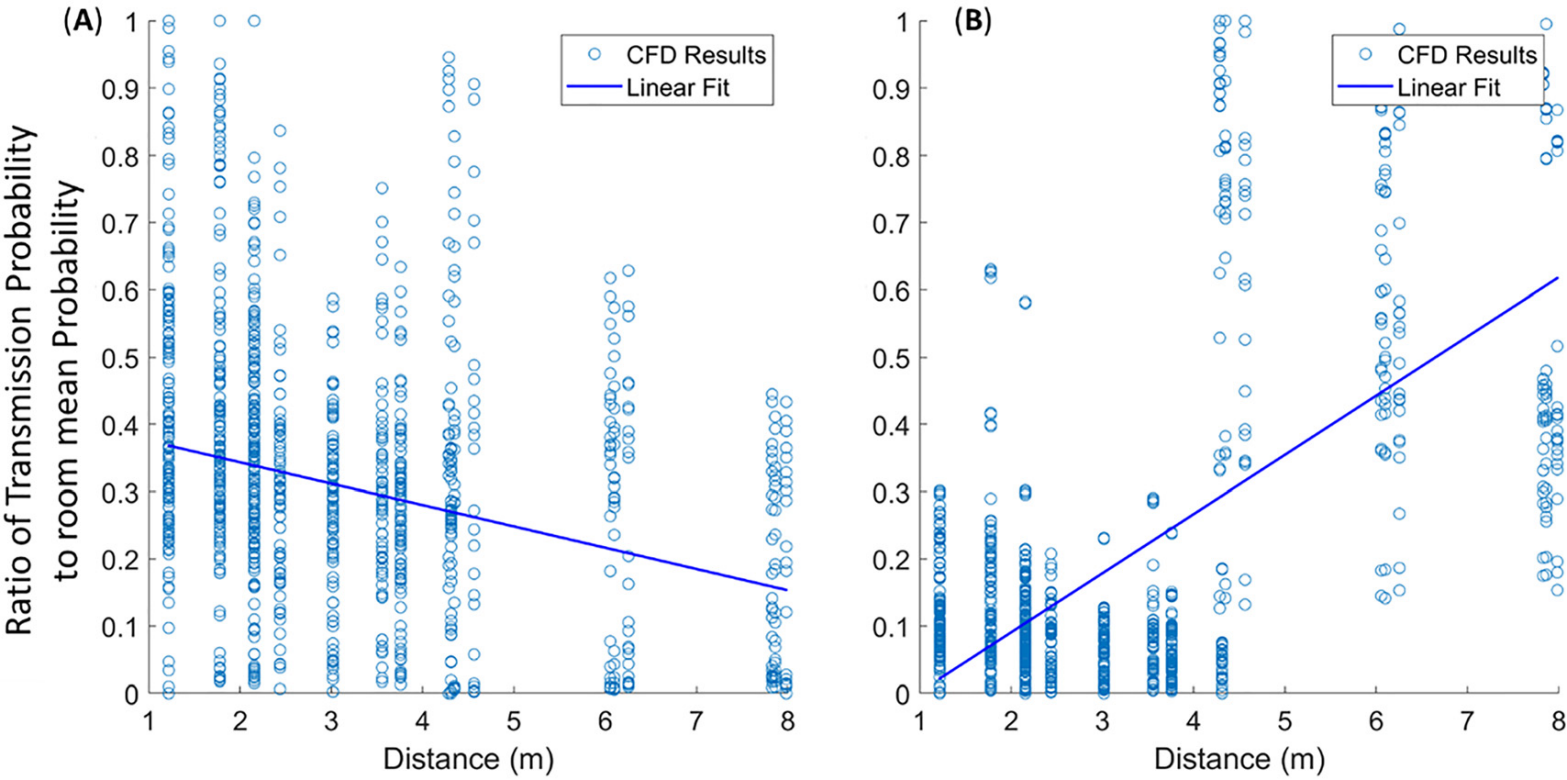
Effect of social distancing to reduce the transmission rate. Each dot indicates the relative transmission risk with respect to the scenario mean (e.g., with/without face coverings, ventilation on/off, etc.) after 60 min. The line is a linear curve fit to the observations. (a) College classroom, where the downward-sloped line indicates a mild reduction in transmission with social distancing. (b) Elementary classroom, which has an upward slope indicating ineffective social distancing. It is critical to note that this elevated risk with distance is associated with the teacher's elevated pulmonary rate.

A college course setting is evaluated with various mitigation methods in Fig. 4(a) using pulmonary and quanta generation rates of adults. The mitigation technologies are indicated on the left, while the statistical representation (box–whisker plot with outliers plotted as symbols, and the line indicating mean) of the transmission probability to the right. The highest risk and variability in transmission rates are the classroom settings that lack ventilation and any mitigation method (∼25% mean with peak routes >40%). This is followed by a group that has active ventilation (∼16% mean with peak routes >30%), which is relatively independent of heating, cooling, or the filter MERV 7 or 11 ratings. Note that with ventilation, the variance in transmission probability is significantly reduced due to filtration and increased dispersion (due to turbulent mixing) throughout the room. The dispersion of quanta of the three highest transmission routes for the no-ventilation, with face-covering, no-desk-shield case is depicted in Figs. 5(a)–5(c). The plots include three-dimensional isosurfaces (upper panel), a contour map of the quanta concentration at the student head level along with highlighted routes (lower panel). These peak routes indicate the formation of cells (stable recirculation zones) within the classroom that tend to trap and lead to elevated concentrations of quanta, leading to increased transmission probability routes. With ventilation, indicated with cooling in Figs. 5(d)–5(f) and heating in Figs. 5(g)–5(i), the cells are reduced in size, which is a large factor driving the reduced transmission probability. The next grouping is associated with the usage of face coverings or food-based source control methods that actively reduce emission of viral particles. Face coverings without ventilation indicated low transmission rates (5%–9% mean, 15% peak), and when utilized with ventilation the mean was roughly the same (5%–7% mean) but the peak dropped (10% peak). When compared to the food-based source control, without ventilation the transmission was roughly 1.5% higher for the mean and peak as compared to the cases with face covering (6.5%–10.5% mean, 11.5%–16.5% peak). Finally, the lowest transmission probability grouping combines mitigation strategies with a combination of face coverings, ventilation, and various air purification strategies (3%–5% mean, peak 8%). Additionally, and in agreement with previous studies,^13^ it is observed that desk shields do not appear to be an effective mitigation strategy. In fact, the desk shields slightly increased the mean and median risks, indicating a minor overall increase in transmission. The desk shields do, however, reduce the peak outlier scenarios with and without face coverings. The simulations indicated that desk shields trap the viral particles, decreasing mixing, and lead to a scenario where they later disperse throughout the room with slightly elevated concentrations. In practice, and well beyond what this study has evaluated, relaxed mask adherence could be another cause of desk shields leading to elevated transmission risk.^13^ The risk with face coverings and desk shields is indicated to be far less than without a mask and desk shields (Fig. 4). Another interesting observation is that, in the context of airborne pathogens, social distancing did not appear to be effective in reducing the transmission probability^14^ and the mitigation factors were much more effective. Figure 6(a) plots transmission probability (normalized by the room mean) vs distance, which indicates an overall decreasing effect of the mean (indicated by the line). These distances were calculated from the CFD student and teacher breathing zone locations; since there are varying distances in each classroom, we can infer the effect of distancing on the exposure results. There are two main features critical to this assessment: (1) fluctuations about the mean tend to be just as high at 4.5 m as they are at 1 m (∼3 ft) based on the WHO guideline^26^ and revised Centers for Disease Control and Prevention (CDC) guidelines^27^—indicating that the CDC social distancing guideline may not be effective enough to impose complications associated with social distancing—and (2) if social distancing was effective, we would anticipate observing a precipitous drop off in the mean or max which does not clearly occur. The increased incidence transmission routes were previously described^14^ and can be described as a direct path formed through complex flow patterns forming between mask baffling, thermal plumes, and ventilation system. In general, viral particles entrained into the HVAC do not lead to increased probability routes as the HVAC leads to improved mixing and more uniform distribution of viral particles in addition to the filtration. The combined results indicate that portable classrooms are not more dangerous than conventional classrooms for the scenarios studied; face coverings and source control approaches are more effective than ventilation alone; combining mitigation strategies is effective; and social distancing did not indicate effectiveness.

To better quantify the data, we can study the underlying mathematics associated with transmission probability from the Wells–Riley model. Previous work established that the Wells–Riley model result correlates well with the mean CFD result.^14^ In the model, transmission to an individual directly correlates with the probability that the person inhales an infectious dose. Dissecting the model reveals that a person will become infected based on their pulmonary rate, the local concentration of the viral particles (HVAC/filtering/source control), and time (fixed at 60 min in these studies). These are the underlying factors that drive transmission, and the goal of mitigation technologies and strategies is to reduce these factors. Per Eq. (4), The transmission events are dictated by the ratio of the inhaled volume of viral particles to the infection dose, or $V_{\text{inhaled}}/V_{\text{quanta}}$. Figure 4(b) provides a plot of this ratio against the transmission probability and displays a clear correlation with the ranked mitigation strategies in Fig. 4(a). In a general sense, mitigation strategies reduce $V_{\text{inhaled}}$ while the infectious dose, $V_{\text{quanta}}$, relates to transmissibility (e.g., in the context of SARS-CoV-2, the Delta strain can either lead to an increase in $V_{\text{inhaled}}$ and/or decrease $V_{\text{quanta}}$ compared to base strains). Considering these mechanisms, the studies are expanded to consider age (which affects pulmonary rate) and the Delta strain of SARS-CoV-2, which is estimated to be 100% more transmissible.^28^

### B. Elementary classroom

**FIG. 7.**
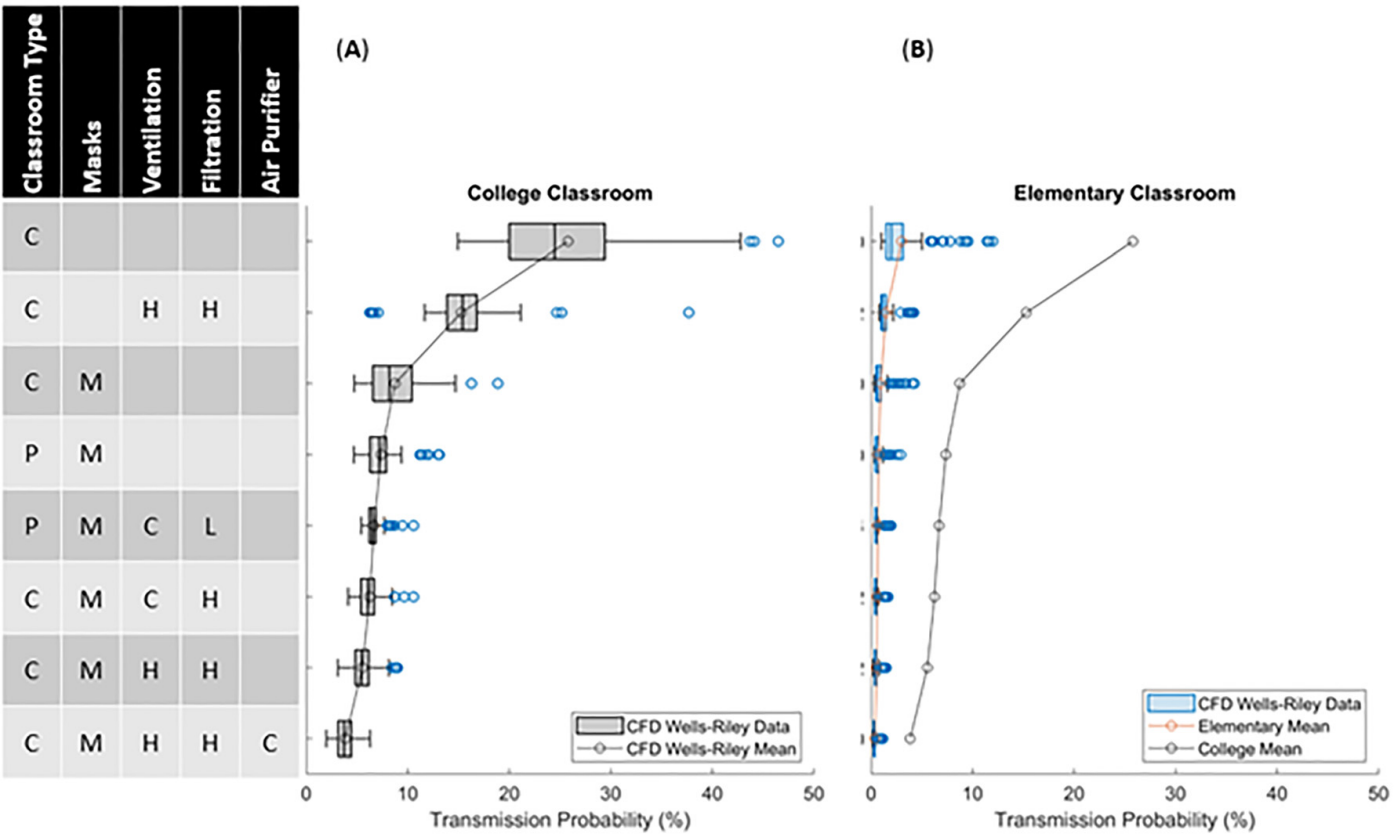
Comparisons of college and elementary transmission probabilities for 90 transmission routes for each setting predicted after a one-hour classroom setting with one infected person. These box-whisker plots use the box to indicate the lower quartile, median, and upper quartile, while the bars indicate the lower and upper standard deviations. The blue dots indicate outliers, while the black dots with the line indicate the mean for each case. (a) College classrooms that are established with adult rates for viral emission and pulmonary rates. (b) Elementary classroom established with child rates for viral emission and pulmonary rates for the nine students, and adult rates for the teacher.

The effort expands the results to an elementary school classroom [Fig. 7(b)] setting. Such evaluations are considered in the same classroom configurations, but using the lower pulmonary and quanta generation rates of 6–11-year-old children (Table I). These elementary school scenarios are compared to identical scenarios in a college setting in Fig. 7(a). Note that this study chose to use a one-hour long class for elementary schools despite the fact that the classroom time periods in elementary school settings can be longer. For example, an eight-hour school day could be composed of three two-hour blocks with breaks for recess, lunch, exercise, art, and/or music. Differences in schools are too complex and variable to capture differences in the scope of this study; hence, we fix the time period to 1 h to enable a comparative analysis of college classrooms (adults). Previous studies have indicated low transmission rates in elementary school settings,^29^ with cases following reopening schools with students aged 6–13 years of age were 0.8% per day as compared to a 1.4% per day for students aged 14–17 years.^30^ This trend has notably occurred globally.^31^ In elementary schools, the students (and not teachers) have decreased pulmonary and viral emission rates, which are reduced by 79.8% and 79.1%, respectively,^23,32^ in children (Table I). In the context of such factors, the risk in elementary school settings is predicted to be substantially lower than the risk in a college room [comparing the college mean line to the elementary school mean line in Fig. 7(b)]. In fact, the mean and median risk from a non-ventilated elementary classroom without any other protocols was lower than the college classroom with the highest number of protocols. Such findings analytically support the empirical evidence that elementary schools are not as prone to airborne transmission as those of older students, which can be directly tied to reducing two factors: (1) emissions of viral particles and (2) inhalation of viral particles. Additionally, for the elementary classroom setting [Fig. 7(b)], there are several outliers that demand further explanation. These outliers are associated with the transmission routes from the teacher's increased viral emission rate relative to elementary students. Additionally, as the model positioned the teacher further from the students, the scenario drives what appears to be an adverse trend with distance that can be observed in the linear fit in Fig. 6(b). The line has a trend that indicates that distance is worse; but these routes, as compared to the declining routes, are associated with the teacher. Such findings, along with increased pulmonary rates during speech, are likely the cause for teachers with face coverings reducing transmission more so than the students.^13^ In general, there is a clear effect associated with age that needs to be considered in in-person schooling.

### C. Delta variant

**FIG. 8.**
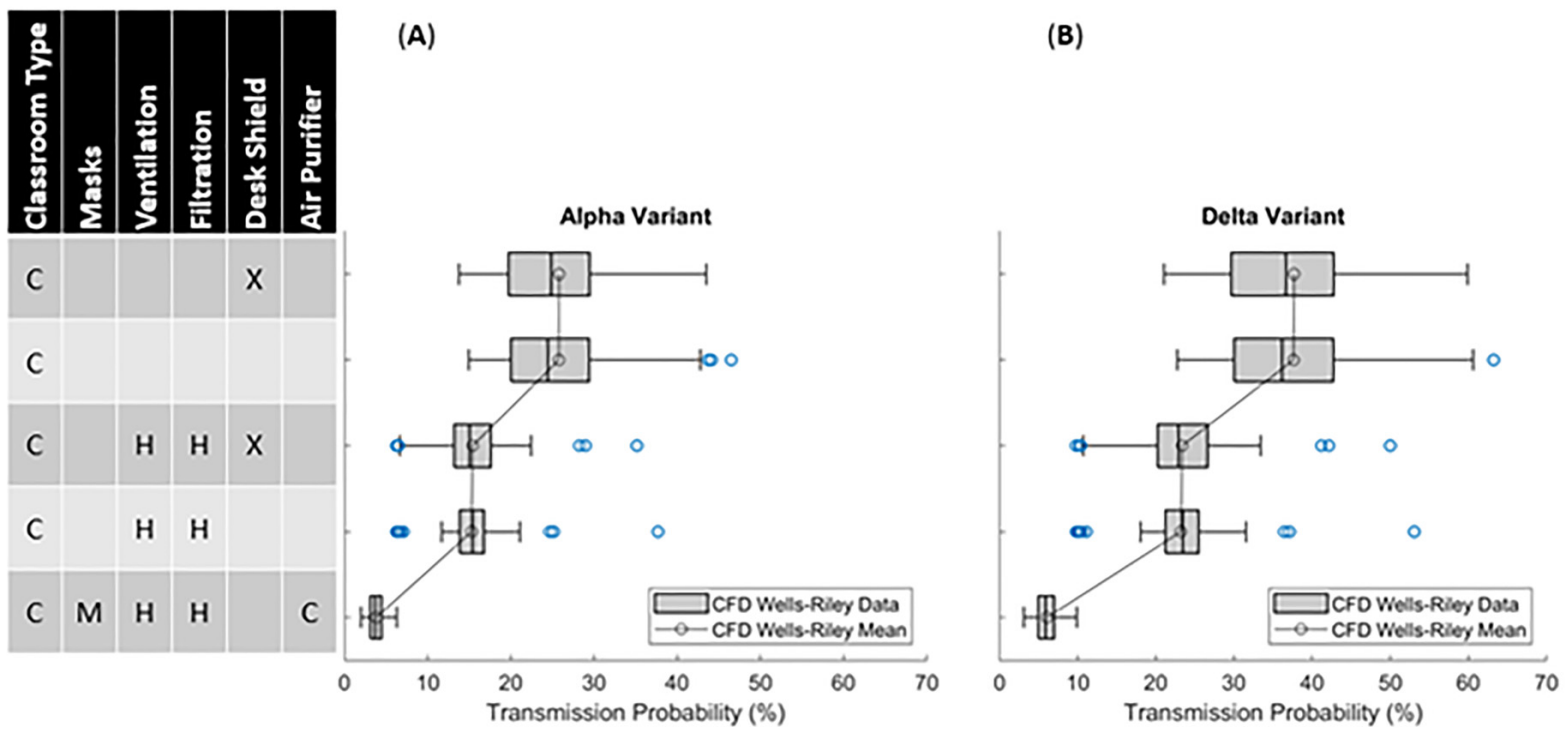
Comparisons of transmission probability statistics for baseline SARS-CoV-2 and an estimation of the Delta variant within a college classroom setting after a one-hour classroom setting with one infected person. These box–whisker plots use the box to indicate the lower quartile, median, and upper quartile, while the bars indicate the lower and upper standard deviations. The blue dots indicate outliers, while the black dots with the line indicate the mean for each case. (a) Using baseline SARS-CoV-2 transmission rates. (b) Using Delta-variant transmission rates.

The second extension of the college classroom aims to compare a subset of mitigation strategies in the context of a model for the base variant of SARS-CoV-2 [Fig. 8(a)] to the more transmissive Delta variant [Fig. 8(b)]. Previous efforts have estimated the increased transmission rates of the Delta variant from 60% (Refs. 28 and 33) to 100% (Ref. 34) with an increase in the viral load of the infected person.^33,35^ Hence, the viral emission rate is increased by 100% (Ref. 34) to approximate the Delta variant. A subtle point that should be emphasized is that the Delta variant is suspected to be associated with an increased quanta emission rate,^33,35^ but transmission rates could also relate to extended airborne life and/or decrease in the infectious dose, which are underlying assumptions in these analyses. The results indicate that the Delta variant can be controlled using several mitigation methods that decrease the transmission rates, which may include face coverings, air purifiers, etc. As indicated in Fig. 8(b), the overall rise in transmission rate is observable; however, it is also manageable with additional mitigation measures. Recall Fig. 4(b), where the transmission probability reduction is highlighted to be a strong function of lingering quanta in the classroom. Hence, a rise in quanta due to the Delta variant is best countered with additional mitigation factors. More specifically, for a baseline classroom without ventilation or mitigation strategies, when exposed to the Delta variant the likely rise in transmission events will range from 50% to 70%. If the same scenario utilizes a ventilation system, the transmission rates would remain consistent. Additionally, if the classroom utilizes all measures (face coverings, ventilation, and purifier), the transmission rates would still decrease from 80% to 90% (with respect to no mitigation measures). The overall analyses indicate that, despite the increased transmissibility of the Delta variant, in-person classroom settings can potentially be manageable with multiple, in-practice, mitigation strategies. Additionally, the previous observation of reduced effectiveness more intervention measures along with no benefit when using more than 7 measures^13^ (e.g., using face coverings, desk shields, and air purifiers is three) may need to be reconsidered in the context of the Delta variant. These observations must also be considered under the premise that school settings are complex use-cases for analysis and demands considering multiple scenarios.

## IV. CONCLUSIONS

In summary, classroom environments demand critical evaluation as schools return to normal operation. This work studied several configurations and mitigation methodologies using CFD methods to support guidance in the return to schooling. Using CFD, we focus on detailed statistics of transmission from the studies. The results help to quantify mean and peak transmission rates in various scenarios.

Confined, indoor settings without ventilation are the most concerning and display the highest demand for face coverings, source control, and/or air purifiers. These mitigation measures should be enforced in combination with other more obvious measures (such as opening windows). When we study details of the transmission events in the context of these scenarios, we find little evidence in the effect of social distancing and barriers, indicating that those measures are not critical. This is especially true in the context of mask mandates. We also studied various classroom types, where we anticipated portable classrooms to be more dangerous. However, we found that these rooms typically have stronger ventilation that prevents transmission events due to increased filtration. Future studies could expand on these cases for larger classrooms, increased students, and positivity rates.

We also studied the effect of age. In the context of the given assumptions, that is, seated students, elementary school-aged students displayed a significantly lower transmission rate than older students. The reason for this decreased transmission rate is due to the students emitting fewer viral particles combined with a lower inhalation rate. Hence, transmission processes are reduced. These factors highlight why, despite children typically spreading disease, children are not likely spreaders of airborne diseases. Additionally, in the context of elementary school settings, there is a significant impact on teacher masking. The results indicate that, in elementary schools, teachers are the most important persons who need to utilize masks. Future studies may want to explore specific age groups for transmission rates, and not combine elementary, middle, and high schools.

Finally, the Delta variant was evaluated. Although it is presently threatening to disrupt plans to reopen schools, we find that enforcing even one additional mitigation mechanism can effectively prevent a scenario that is worse than with the Alpha variant. As more information develops about transmission mechanisms from the Delta variant (and future variants), we can better prepare classrooms and other environments.

## Data Availability

The data that support the findings of this study are available within the article.
